# Critical Hemorrhage Caused by a Size-Mismatched Extracorporeal Membrane Oxygenation Cannula in a Patient with Myotonic Dystrophy Type 1: A Case Report and Literature Review

**DOI:** 10.3390/medicina60060969

**Published:** 2024-06-12

**Authors:** Changsik Shin, Kwon Cheol Yoo, Dae Hoon Kim

**Affiliations:** 1 Department of Surgery, Uijeongbu Eulji Medical Center, Eulji University, Uijeongbu 11759, Republic of Korea; 2 Department of Surgery, College of Medicine, Chungbuk National University, Cheongju 28644, Republic of Korea; 3 Department of Surgery, Chungbuk National University Hospital, Cheongju 28644, Republic of Korea

**Keywords:** ECMO, size mismatch, vascular closure device, case report, myotonic dystrophy type 1

## Abstract

*Background and Objective*: Although extracorporeal membrane oxygenation (ECMO) is an essential life-saving technique for patients with refractory cardiopulmonary shock, it can be fatal in certain cases. *Case Presentation*: A 19-year-old girl treated with ECMO presented with acute limb ischemia 2 days after cannula removal. The decannulation was performed percutaneously by an interventional cardiologist, and the vascular surgery department was consulted after the patient developed symptoms. The first suspected diagnosis was thrombosis due to incorrect use of the closure device. However, the artery had ruptured due to the insertion of a catheter with a cannula that was larger than the patient’s artery. *Management and Outcome*: Fortunately, excessive bleeding due to the size-mismatched cannula was prevented by an unintentional complication of the closing device, which saved the patient’s life. She underwent a right common femoral artery thrombectomy and patch angioplasty. Hospital guidelines have changed regarding the surgical removal of ECMO cannulas. *Discussion*: This report aims to highlight the importance of two aspects that are critical to a successful outcome: individualized cannula selection followed by precise insertion and removal and postoperative evaluation of a patient’s final status.

## 1. Introduction

Extracorporeal membrane oxygenation (ECMO) is a crucial life-saving intervention in patients with refractory cardiopulmonary shock [1,2,3]. For cannulation, either a percutaneous method or an open cutdown approach is used; the former is preferred due to the need for emergent cannulation and the ease of approach [4,5]. Herein, we report a unique case in which an artery ruptured due to a cannula size mismatch. This complication could have been fatal, as it caused excessive bleeding. However, fatality was prevented by the unintended complication of thrombotic occlusion due to vascular closure devices.

## 2. Case Report

A 19-year-old girl who visited the emergency ward because of a sudden loss of consciousness and accompanying cardiac arrest was administered cardiopulmonary resuscitation. She later reported a family history of myotonic dystrophy type 1. She required emergency percutaneous venoarterial ECMO owing to a low cardiac output and sustained ventricular fibrillation on electrocardiography. Catheters were inserted through the right common femoral artery (CFA) and the left common femoral vein (CFV). After 3 days of ECMO maintenance, the catheters were removed percutaneously by an interventional cardiologist. At the time of catheter removal by the intervention cardiologist, the catheter was removed with compression of the proximal femoral artery to minimize bleeding. The intervention cardiologist noted that the removal was accomplished with no more bleeding than usual. The main catheter inserted into the right CFA was relatively large, at 16.5 Fr; therefore, a Perclose Proglide suture-mediated closure device (Abbott Vascular Devices, Redwood City, CA, USA) was used for artery closure. The catheter used for distal protection was relatively small, at 6.5 Fr; thus, a FemoSeal™ Vascular Closure System (St. Jude Medical, St. Paul, MN, USA) was used.

**Figure 1:**
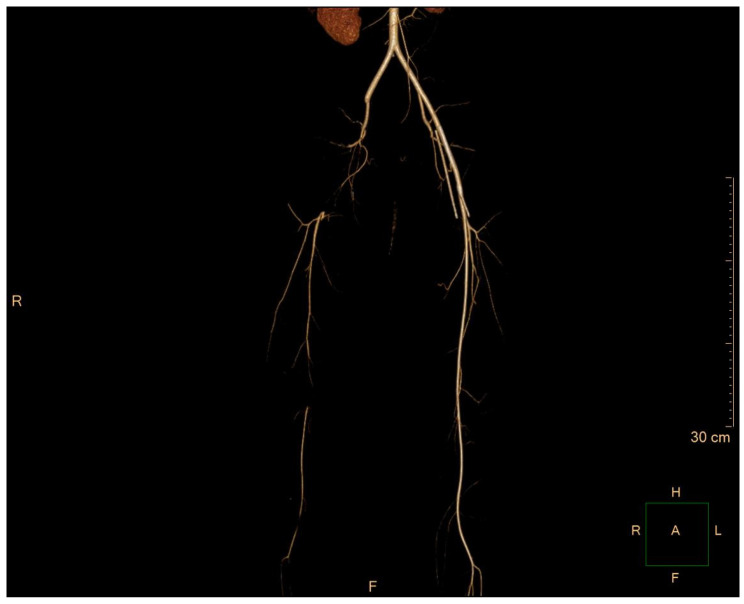
Lower-extremity angio CT was performed following the patient’s complaints of ischemic symptoms.

When the sedative drug used to control the patient’s irritability was discontinued 2 days after catheter removal and she was able to communicate, she complained of decreased movement and pain in her right leg. Her leg was cold to the touch and had lost motor function altogether, with significantly reduced sensation compared to the contralateral side. Because the patient had been on ECMO 2 days prior, she was being administered intravenous heparin (with a target-activated partial thromboplastin time (aPTT) of 60 to 80 s for therapeutic anticoagulation). The patient underwent computed tomography angiography (CTA) under the suspicion of acute limb ischemia, which revealed thrombotic occlusion from the right external iliac artery (EIA) to the superficial femoral artery (SFA) (Figure 1).

Following the CT scan, the patient was referred to the department of vascular surgery for further investigation and treatments. Firstly, the ischemic symptoms were thought to be due to complications associated with the vascular closure device during the removal of the catheter. A preoperative ultrasonic examination revealed the presence of a thrombus in the artery between the vascular closure device’s gaps. The blood flow in the distal arteries of the leg was observed to plateau, despite the absence of any obvious thrombus. Surgery was planned with angioplasty under the suspicion of eccentric compression of the lumen of the artery due to incorrect use of the Perclose ProGlide™. It was determined that local anesthesia would be used rather than general anesthesia in order to minimize the time of ischemia of the lower limb.

**Figure 2:**
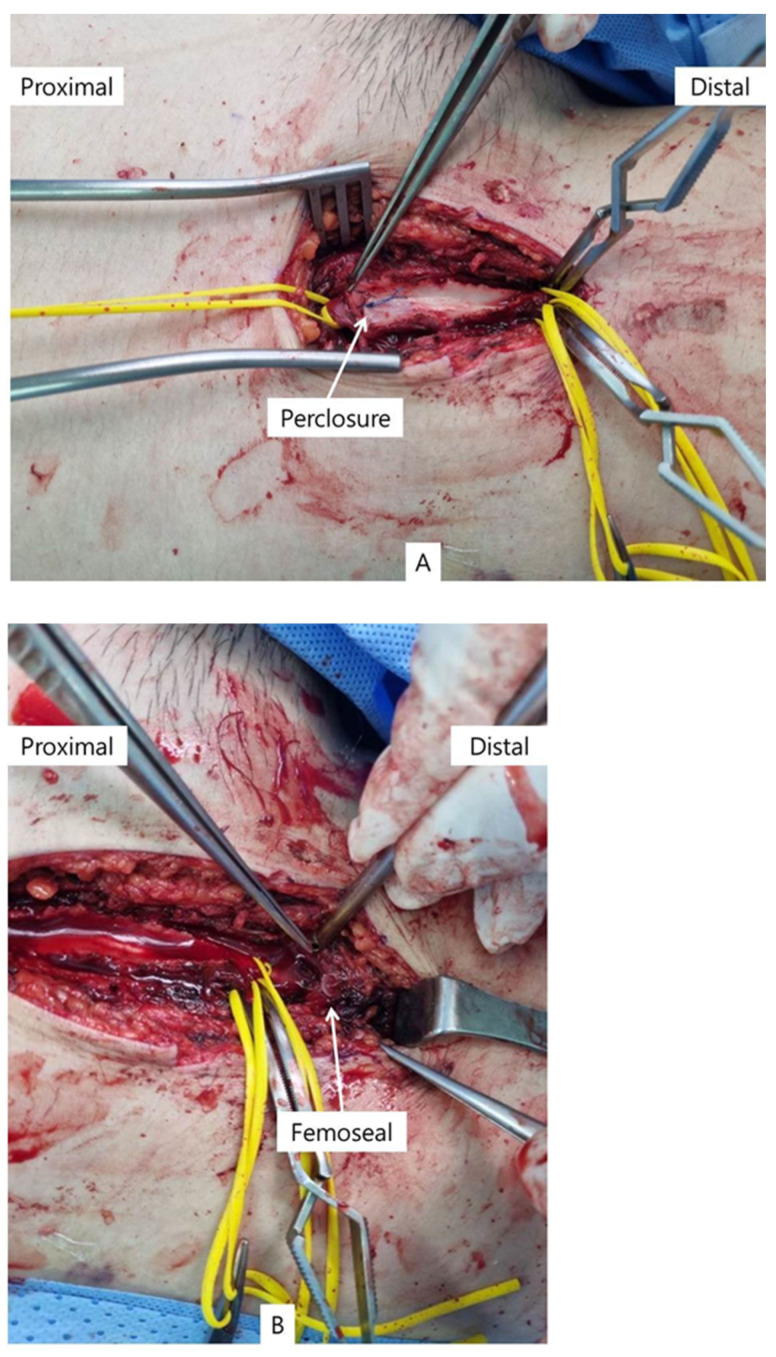

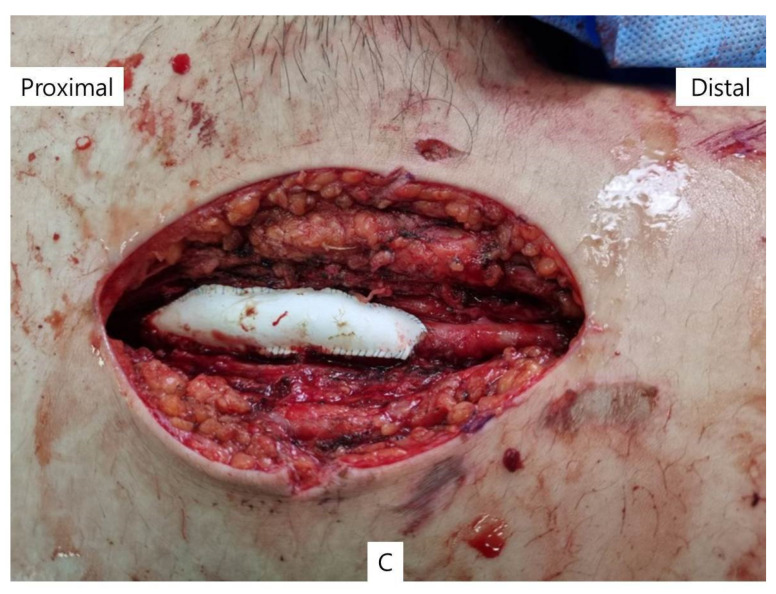
(**A**) Ruptured common femoral artery with clamped by Perclosure. (**B**) Ruptured common femoral artery with clamped by Femoseal. (**C**) Patch angioplasty with PTFE graft.

A longitudinal incision of approximately 15 cm was made along the femoral artery. Contrary to expectations before surgery, as shown in the operative field in Figure 2, the right CFA had ruptured to a length of approximately 10 cm. Moreover, intraluminal occlusion was caused by suturing the entire vessel of the proximal CFA through the anterior wall of the artery to the posterior wall by Perclose ProGlide™ and the proximal SFA by FemoSeal™. After withdrawing all incorrectly inserted closure devices, a thrombectomy was performed for both the proximal and distal arteries. It was possible to observe the presence of blood flow in both the proximal and distal parts of the artery. A patch angioplasty was performed on the ruptured artery using polytetrafluoroethylene, as it was not possible to harvest autologous blood vessels large enough to repair the rupture. A triphasic flow pattern was observed in all the right leg arteries postoperatively. The patient was then administered apixaban (5 mg) instead of a heparin injection. One year after surgery, the patient is now undergoing rehabilitation without complications. In the wake of this case, surgical removal of the ECMO cannula has become standard practice in hospitals.

## 3. Discussion

ECMO plays an important role in treating patients with reversible lung and heart failure. Depending on the organ to be supported, it is divided into venoarterial and venovenous ECMO, and the type of vessel to be approached is determined. ECMO complications can occur in the circuits or in the patient, occasionally resulting in fatality. Relatively large-sized cannulas are required to maintain ECMO function and iatrogenic cannula-site hemorrhages have been reported to occur in 26.7–31.4% of patients [6]. Retroperitoneal hemorrhage may occur during the cannulation of tortuous or small-sized vessels [7]. Conversely, it is interesting to note that thromboembolic events, including deep vein thrombosis, pulmonary embolism, and cannula occlusion, have been reported in 2–9% of cases [6,8,9].

In this case, the patient was first suspected to have suffered from a thrombotic occlusion owing to the incorrect usage of the closing devices; however, the situation was found to be completely different intraoperatively. The diameter of the recovered patient’s CFA was 5.5 mm on a CT scan performed at the time of the vascular surgeon’s initial contact, and given the state of cardiac arrest at the time of cannulation, the artery was estimated to be smaller than 5.5 mm. Therefore, a 16.5 Fr (5.5 mm) diameter cannula would have been relatively large for the size of the vessel. During the initial procedure, the insertion of a catheter larger than the diameter of the vessel would have resulted in a rupture, which could have been fatal due to the excessive bleeding that would have occurred during removal. The removal of the catheter from the ruptured artery could have resulted in significant bleeding. However, the potentially fatal situation was averted through occlusion of the vessel, which is a complication of vascular closure devices.

In the case of arterial damage caused by percutaneous catheterization, there is disruption to the continuity of the arterial wall, which may exacerbate the extent of damage due to the pulsatile pressure exerted on the artery. In the most severe cases, this may result in the formation of a hematoma, a pseudoaneurysm, or even a rupture at the vascular access site. Catheterization-related pseudoaneurysms are a rare occurrence, with an incidence of 2–7%. The majority of these pseudoaneurysms can be successfully treated with manual or probe compression or thrombin injection. The femoral artery is the most commonly reported location.

A summary of previous cases reporting rupture of catheterization-induced pseudoaneurysms is presented in Table 1. Rupture of pseudoaneurysms caused by catheterization appears to be uncommon, with the exception of cases involving other orthopedic surgery, trauma, drug abuse, and infectious aneurysms.

It appears that the majority of cases occur in older patients who are undergoing anticoagulation therapy.

In addition, underlying medical conditions can also be risk factors for artery ruptures. Myotonic dystrophy (DM) type 1 occurs when a gene on chromosome 19 called DMPK contains an abnormally expanded region near the regulation region of another gene, SIX5. DM type 1 causes cardiac conduction abnormality, cataract, weakness, and wasting of voluntary muscles in the face, neck, and lower arms and legs [18].

The patient described in this case is an underweight female, 164 cm tall, weighing 45 kg, with a family history of DMPK mutations in her maternal grandfather, uncle, mother, and sister. The patient had been experiencing progressively worsening limb weakness for approximately one year prior to the admission. The cardiac arrest that led to the emergency room visit was caused by a heart problem: DM type 1. DM type 1 also affects blood vessels and is thought to be associated with dysfunction of smooth muscle in the vessel wall, which may lead to structural instability and complications [19,20]. Additionally, the patient’s lack of exercise, which is believed to have progressed rapidly over the past year, may have affected the distensibility and elasticity of the artery, causing it to fail to maintain its shape during catheterization and ultimately leading to a rupture [20,21,22].

The “gold standard” for vascular access closure is manual compression. However, this method is time-consuming and requires prolonged bed rest for the patient to achieve a perfect closure. With the introduction of vascular closure devices in the early 1990s, the reliance on manual compression has decreased. A systematic review published by Noori in 2018 found that the use of vascular closure devices did not result in a meaningful difference in complications or safety compared to manual compression (12% for vascular closure devices vs. 13% for manual compression). Nevertheless, vascular closure devices should be used with caution, as infections (0.6% vs. 0.2%) and thrombosis (0.3% vs. 0%) can be fatal to patients.

In order to prevent a recurrence of such unfortunate incidents, it is of the utmost importance to conduct a comprehensive investigation into the circumstances that led to their occurrence. The situation is likely to be urgent and, in most cases, every minute counts at the time of ECMO cannulation. Firstly, it is necessary to identify the cause of the blood vessel rupture, which in this case was a result of a size mismatch. The dimensions of blood vessels exhibit considerable variability, extending beyond the influence of race to encompass gender and body size. Consequently, it is imperative to adhere to the principle of individualized assessment when dealing with such issues, rather than relying on generalized guidelines. In the event of unstable vital signs, the diameter of the artery intended for insertion may appear significantly smaller than it would under normal circumstances. In order to minimize the risk of error during insertion, it is essential to accurately measure the size of the insertion site and to ensure that the cannula to be inserted is appropriately sized.

Secondly, in the case of a vascular closure device, the puncture site is repaired by collecting and repairing the hole, and the lumen of the blood vessel must be filled with blood for normal functioning. However, if the blood vessel is ruptured or the vascular closure device is inserted relatively deeply or shallowly, the hole may not narrow, causing bleeding, or the back and front walls of the blood vessel may bind together, resulting in ligation of the blood vessel. It is presumed that a ruptured vessel resulted in a lack of blood in the lumen, which in turn caused the vascular closure device to operate with the device deeply inserted. This is believed to be the cause of the complication in this case.

Thirdly, it is crucial to emphasize the significance of the post-cannulation procedure. The current hospital standard is to remove the ECMO cannula surgically. However, if we consider the situation in retrospect, the percutaneous removal procedure does not provide direct visual confirmation of proximal or distal blood flow. However, the palpation of a distal artery (e.g., the popliteal artery, dorsalis pedis artery, or posterior tibial artery in the ankle) to check for a pulse, or the measurement of blood flow via ultrasound immediately after the procedure, would have identified the problem more promptly. The optimal treatment plan for this particular situation would have been implemented at the appropriate time.

To the best of our knowledge, vascular rupture due to size mismatch and vascular occlusion due to complication of a vascular closure device have been reported separately; however, this is the first time that a simultaneous occurrence has been reported, which turned out to be fortunate for the patient.

## 4. Patient Perspective

The patient was grateful for the successful outcome and had no discomfort. Moreover, no motor or sensory complications have been observed thus far. As a consequence of the prolonged period of intensive care unit treatment, there was a significant loss of muscle mass, and following one year of rehabilitation, the patient was discharged from the hospital with the ability to walk with the aid of a brace.

## 5. Conclusions

ECMO, which is widely used to treat patients with cardiopulmonary arrest, requires a high level of technical expertise. It is important to note that the vascular status of patients may vary depending on their underlying disease. It is crucial to understand the individualized and precise measuring and removal of the cannula to reduce postoperative complications and to assess the postoperative status of the patient promptly.

## Figures and Tables

**Table 1:** Summary of case reports for catheterization-related femoral artery rupture, with other causes excluded.

Authors (y)	Sex/Age	Symptom	Location	Time	Catheter Size	Rupture Size	Cause	Medication History	Treatment
Jones et al. (1992) [10]	M/63	Swelling and bleeding from inguinal area	Right Common femoral artery	7 days	Unreported	1.5 × 2.2	Cardiac angiography	Full-dose heparin -> Coumadin	Open repair
	M/57	Pain with swelling and bleeding from inguinal area	Right Common femoral artery	5 days	Unreported	1.0 × 1.0	Cardiac angiography	Aspirin, Coumadin, Dipyridamole	Open repair
	M/50	Swelling and bleeding from inguinal area	Right Common femoral artery	4 days	Unreported	1.4 × 1.5	Cardiac angiography	Full-dose heparin	Open repair
Ma et al. (2005) [11]	W/65	Abdominal wall hematoma	Right Common femoral artery	Unreported	5 French	13 × 18	Percutaneous closure of an atrial septal defect	Enoxaparin -> Warfarin	Open repair
Renner et al. (2013) [12]	W/79	Shock with pain	Right Superficial femoral artery	17 days	5 French	23 × 20	Arterial monitoring catheterization	Warfarin, Corticosteroid	Open repair
Petrou et al. (2015) [13]	W/69	Shock with pain	Right deep femoral artery	6 h	6 French	12 × 8.5	Cardiac angiography	Unreported	Open repair
Kulkarni et al. (2016) [14]	W/75	Shock	Right Common femoral artery	10 days	Unreported	11.5 × 7.5	Venous catheterization for hemodialysis	Heparin	Open repair
Patrick et al. (2019) [15]	M/69	Shock with swelling	Left Common femoral artery	7 years	Unreported	8.2 × 7.9	Lower-extremity angioplasty	Aspirin, Clopidogrel	Open repair
Wygant et al. (2020) [16]	W/60	Pain and bleeding from inguinal area	Right Common femoral artery	2 years	Unreported	3.2 × 4.0	Lower-extremity angioplasty	Unreported	Untreated due to death
Ok et al. (2024) [17]	M/90	Shock with mental change	Right Superficial femoral artery	3 days	8 French	4.1 × 2.8	Cerebral angiography	Aspirin, Cilostazole	Open repair

## Data Availability

The original contributions presented in the study are included in the article. Further inquiries can be directed to the corresponding author.
